# Indium Tin Oxide-Based Voltammetric Biosensor for the Detection of Antibodies Against the SARS-CoV-2 Virus Spike Protein

**DOI:** 10.3390/s25216737

**Published:** 2025-11-04

**Authors:** Greta Zvirzdine, Maryia Drobysh, Almira Ramanaviciene, Vilma Ratautaite, Sarunas Zukauskas, Migle Stanciauskaite, Ieva Plikusiene, Arunas Ramanavicius

**Affiliations:** NanoTechnas—Center of Nanotechnology and Materials Science, Faculty of Chemistry and Geosciences, Vilnius University, Naugarduko Str. 24, 03225 Vilnius, Lithuania; greta.zvirzdine@ftmc.lt (G.Z.); mariadrobysh@gmail.com (M.D.); almira.ramanaviciene@chf.vu.lt (A.R.); vilma.ratautaite@ftmc.lt (V.R.); sarunas.zukauskas@ftmc.lt (S.Z.); migle.stanciauskaite@chgf.stud.vu.lt (M.S.); ieva.plikusiene@chgf.vu.lt (I.P.)

**Keywords:** SARS-CoV-2 virus, recombinant spike protein, affinity interaction, immune complex formation, antigen–antibody binding, indium tin oxide (ITO) electrode, square wave voltammetry (SWV), ellipsometry

## Abstract

This study aims to propose a plausible application of a novel electrochemical biosensing system for detecting antibodies against SARS-CoV-2 (anti-rS) in serum samples. The uniqueness of this study lies in the biosensor utilizing recombinant spike glycoprotein (SCoV2-rS) immobilized on an indium tin oxide (ITO) electrode modified with (3-aminopropyl)triethoxysilane (APTES). The electrochemical performance was evaluated using square wave voltammetry (SWV), demonstrating a linear relationship between the current density and anti-rS concentration. The limit of detection (LOD) was found to be 113 ng/mL (0.75 nM), and the limit of quantitation (LOQ) was equal to 338 ng/mL (2.25 nM). The reported electrochemical biosensor offers a straightforward and efficient method for evaluating the immune status of individuals who have recovered from COVID-19 and been vaccinated against this virus without the need for any redox probe.

## 1. Introduction

The Severe Acute Respiratory Syndrome Coronavirus 2 (SARS-CoV-2), the causative agent of Coronavirus Disease 2019 (COVID-19), continues to mutate and spread. Thus, the development of methods for the accurate and rapid diagnosis of COVID-19, as well as the monitoring of the immune status of recovered and/or vaccinated individuals, remains a pressing issue. SARS-CoV-2 is an enveloped virus with a lipid bilayer produced by the membrane of the host cell, consisting of the membrane, envelope proteins, and spike glycoprotein (SCoV2-S) [1]. The SCoV2-S are observed as projections from the envelope’s exterior and are crucial for host cell identification, fusion, and viral entry [2]. The human immune system response to SARS-CoV-2 is largely mediated by antibodies, and the SARS-CoV-2-S is a prominent target for neutralizing antibodies [2,3].

Biosensors have been widely used for SARS-CoV-2 diagnostics, showing express, straightforward, and reliable recognition. Numerous studies have been dedicated to detecting the SARS-CoV-2 genome, structural proteins, and expressed antibodies against the coronavirus employing biosensors [4]. These biosensors are mainly based on optical [5,6,7,8], field-effect transistor [9,10,11], colorimetric [12], surface plasmon resonance [13,14,15,16], and electrochemical methods of analysis [17]. Electrochemical biosensors are considered sensitive, simple to operate, rapid, and cost-effective. Methods such as electrochemical impedance spectroscopy [18,19,20], cyclic voltammetry (CV) [21,22,23], differential pulse voltammetry (DPV) [24,25,26], amperometry [27,28,29], and square wave voltammetry (SWV) [30,31] are generally used to obtain an analytical signal. CV is an effective technique for characterizing various electroactive species and correlating the current-potential properties of each engaged oxidation or reduction reaction. SWV is also used in biosensor design due to its high selectivity and sensitivity [32]. The can be utilized to analyze reversible and irreversible reactions, slow electron transfer reactions, and catalytic reactions [33]. Additionally, compared to other pulsed techniques, it can preserve electroactive particles [34]. As the consumption of electroactive particles is reduced, electrode surfaces tend to be less contaminated with non-electroactive products [33]. Applying SWV, there is no necessity to exclude oxygen from the analyte solution, as far as oxygen reduction is incorporated into the background flow [35]. In this method, the applied potential is more cathodic than that for the reduction of oxygen. Notably, SWV is a rapid technique. A complete voltammogram can be recorded within a few seconds, compared to 2–3 min for DPV. Additionally, SWV is more sensitive than DPV, as both forward and reverse currents are measured in the former, whereas only forward currents are measured in the latter [36].

Indium tin oxide (ITO) is considered one of the most widely applied transparent conductive oxide thin films in biosensor design, owing to its good electrical conductivity and optical transparency [37]. Immobilization of biorecognition elements on the ITO electrode surface is a crucial step in the design process of biosensors. Commonly, self-assembled monolayers (SAMs) are applied for the functionalization of the working surface. Owing to straightforward preparation steps, reproducibility, and stability, silane-based SAMs are widely used for the modification of ITO [38]. Here are examples of some silanization agents, namely, triethoxymethylsilane, (3-isocyanatopropyl) triethoxysilane [39], carboxyethylsilanetriol [40], 3-mercaptopropyl trimethoxysilane [41], and (3-aminopropyl)triethoxysilane (APTES) [42]. APTES consists of three hydrolysable ethoxy groups forming Si–O bonds on the top of the ITO electrode surface and a terminal NH_2_ group providing binding with biomolecules, namely, proteins [43].

To date, several studies have focused on the development of biosensors utilizing ITO as a conductive substrate for immobilizing capture biomolecules or detecting target compounds in the diagnosis of COVID-19. Among them, there are spectroelectrochemical [44], impedimetric [45], and dual-gate oxide semiconductor thin-film transistor-based [46] systems for the detection of recombinant spike glycoprotein (SCoV-S); a photoelectrochemical immunosensor for the recognition of SARS-CoV-2 nucleocapsid protein [47]; and electrochemiluminescence detection of antibodies against SARS-CoV-2 [48].

In this work, we report an electrochemical biosensing system for the recognition of antibodies against SARS-CoV-2 in real samples. SCoV2-rS was used as a sensing biomolecule, immobilized on the ITO working electrode functionalized with APTES. The signal registration after interaction between SCoV2-rS and antibodies against SARS-CoV-2 (anti-rS) was performed by CV and SWV. The analytical performance of the system was evaluated by calculating the limits of detection (LOD) and quantification (LOQ).

## 2. Materials and Methods

### 2.1. Chemicals and Other Materials

Indium tin oxide (ITO) coated glass slides with surface resistivity of 12 Ω/cm^2^ were purchased from Sigma-Aldrich (Saint Louis, MO, USA). Ethanol (EtOH) (99.9%, CAS# 64-17-5), (3-aminopropyl)triethoxysilane (APTES) (≥98%, CAS# 919-30-2), Na_2_HPO_4_ (≥99.0%, CAS# 7558-79-4) and NaH_2_PO_4_ (≥99.0%, CAS# 7558-80-7) for preparing phosphate buffer (PBS) solution and glutaraldehyde solution (GA) (50% *w*/*w* H_2_O, CAS# 111-30-8) were obtained from Sigma-Aldrich (Steinheim, Germany). Baltymas (Vilnius, Lithuania) synthesized the SARS-CoV-2 recombinant Spike protein (SCoV2-rS) [18]. A volunteer who received one dose of the Vaxzevria vaccine and tested positive for COVID-19 two weeks later provided serum samples containing antibodies against the SARS-CoV-2 spike protein (anti-rS) at a laboratory of Tavo Klinika, Ltd. (Vilnius, Lithuania) under the Lithuanian Ethics Law (confirmed by the Vilnius Regional Biomedical Research Ethics Committee). Deionized water was used to prepare the aqueous solution. For the preparation of protein and antibody solutions as well as for electrochemical measurements, 0.1 M PBS solution, pH 7.4, was used.

### 2.2. ITO Surface Modification with APTES

**Figure 1:**
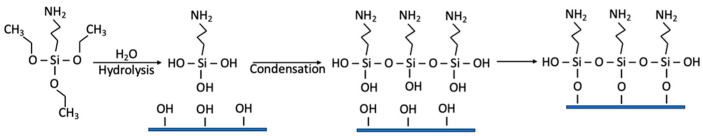
The APTES hydrolysis, condensation, and positioning on the ITO surface result in the formation of ITO/APTES. Adapted [50].

**Figure 2:**
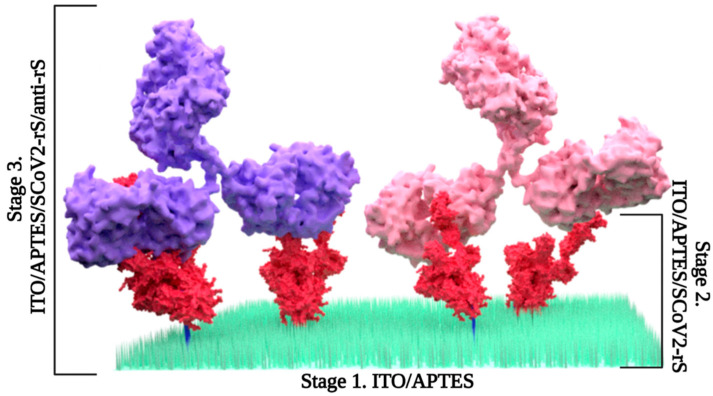
Schematic representation of the experimental stages. Stage 1—formation of SAM resulting in ITO/APTES; stage 2—incubation with SCoV2-rS with formation of ITO/APTES/SCoV2-rS; stage 3—anti-rS coupling resulting in ITO/APTES/SCoV2-rS/anti-rS formation.

In order to activate the ITO surface [49], ITO slides were treated with argon plasma by Super Cool Sputter Coater Leica EM SCD050 (Leica Microsystems GmBH, Praha, Czech Republic) under a vacuum depth of ~0.1 mbar in 5 min. Furthermore, the slides were incubated in a 1% ethanol solution of APTES overnight at room temperature, resulting in the formation of ITO/APTES (Figure 1 and Figure 2, stage 1). After incubation, the ITO/APTES slides were rinsed with EtOH and dried by heating to 40 °C for 30 min on a magnetic stirring hotplate from Heidolph MR Hei-Tec (Schwabach, Germany).

### 2.3. ITO and ITO/APTES Surface Characterization

ITO and ITO/APTES electrode surfaces were characterized by means of electrochemical and optical methods, i.e., CV and ellipsometry. The electrochemical properties of ITO and ITO/APTES electrodes were evaluated using a potentiostat, PalmSens4, controlled by PSTrace5 software, version 5.10.5604 (The Netherlands). The three-electrode electrochemical system consisted of ITO or ITO/APTES as the working electrode, the Ag/AgCl (3 M KCl) electrode as the reference electrode, and a platinum wire as the counter electrode.

Electrochemical measurements were performed to characterize the ITO and ITO/APTES surfaces in a 10 mL electrochemical cell containing a PBS solution, pH 7.4, and 2.5 mM of the redox probe K_4_[Fe(CN)_6_]/K_3_[Fe(CN)_6_]. The redox probe was used only for surface characterization measurements. Potential cycling was performed in the range from −0.1 V to +0.6 V at a scan rate of 0.1 V/s and a step size of 0.005 V. In total, 5 potential cycles were applied to characterize the surface. The 5th cycle was accepted as characteristic of the system.

The ellipsometric experiments were conducted using a rotating compensator-based ellipsometer, J. A. Woollam M2000X (Lincoln, NE, USA), operating in a spectral range from 210 nm to 1000 nm. The angle of incident light was fixed at 70 deg. Ellipsometric parameters Ψ and Δ dependence on wavelength were registered before and after modification using *N*-(3-aminopropyl)triethoxysilane (APTES, 99%).

### 2.4. Immobilization of SCoV-2

ITO/APTES were divided into nine separate sites with an area of 0.196 cm^2^ by means of acrylic double-sided mounting tape placed on the top of the slide. Afterwards, each site was covered with 10 µL of 10 µg/mL SCoV2-rS in the PBS solution and left to air-dry for ~90 min. Furthermore, a custom-built construction with nine cells, each 400 µL in volume, was attached to the sticker. The next step was to covalently bind SCoV2-rS with ITO/APTES. For this purpose, ITO/APTES was placed in a 25 mL beaker with 25% GA solution and incubated in vapor for 15 min. After incubation in GA vapor, the resulting ITO/APTES/SCoV2-rS (Figure 2, stage 2) was kept in a 0.1 M PBS solution, pH 7.4, overnight at 2 °C.

### 2.5. Coupling with Anti-rS

ITO/APTES/SCoV2-rS was rinsed with 0.1 M PBS solution, pH 7.4, and dried with compressed air flow. Further, each cell was covered by 10 μL of anti-rS solution in a range of concentrations from 0 to 200 ng/mL, prepared in 0.1 M PBS solution, pH 7.4, in 10 min at room temperature, eventually forming ITO/APTES/SCoV2-rS/anti-rS (Figure 2, stage 3). After that, each cell was rinsed with 0.1 M PBS solution, pH 7.4, and electrochemical measurements were performed.

### 2.6. Electrochemical Measurements

The binding of the SCoV2-rS was evaluated in a three-electrode electrochemical cell consisting of an ITO-coated glass slide as the working electrode, an Ag/AgCl (KCl 3 M) electrode as the reference electrode, and a Pt wire serving as the counter electrode, with a volume of the electrochemical cell of 50 µL. SWV measurements were performed in a 0.1 M PBS solution, pH 7.4, by the μAUTOLAB TYPE III/FRA2 potentiostat (Metrohm, Barendrecht, The Netherlands) controlled by Nova software package version 2.1.6 from ECO-Chemie (Utrecht, The Netherlands). SWV signal was registered for ITO/APTES/SCoV2-rS/anti-rS formed after treatment with anti-rSpike in a concentration range from 0 to 200 ng/mL. SWV measurements were performed with the following parameters: a potential window from −0.6 to +0.3 V, a step size of 0.005 V, a modulation amplitude of 0.025 V, and a frequency of 20 Hz.

## 3. Results and Discussion

**Figure 3:**
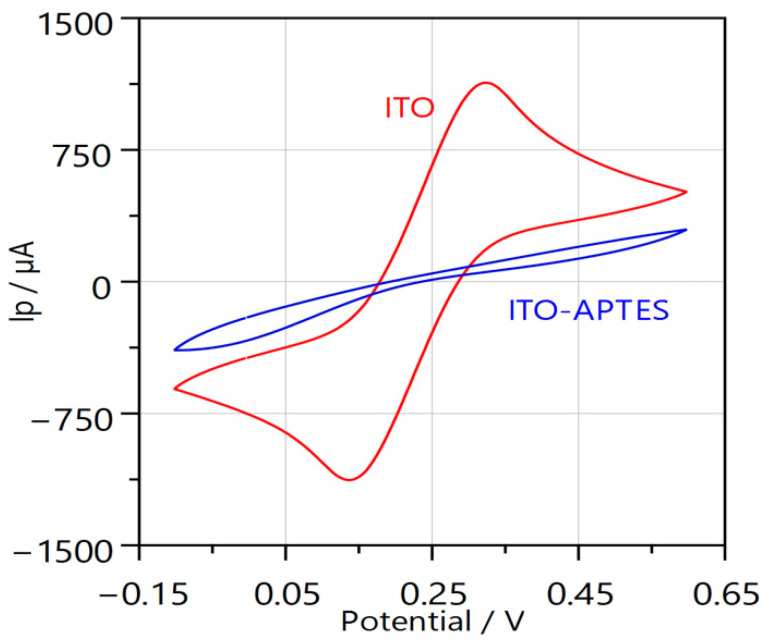
ITO and ITO/APTES surface characterization. Cyclic voltammetry-based surface characterization. CV was registered by potential cycling from −0.1 V to +0.6 V at a scan rate of 0.1 V/s and a step size of 0.005 V. In total, 5 potential cycles. This figure demonstrates the 5th potential cycle.

**Figure 4:**
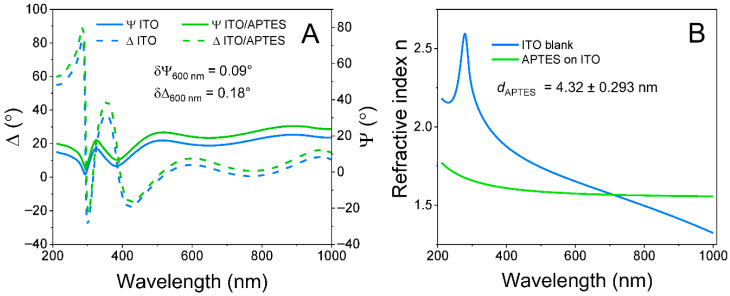
Ellipsometric measurement and modeling of ITO and APTES-functionalized ITO layers. (**A**) Ellipsometric parameter Ψ (solid line) and Δ (dashed line) shifts before ITO modification (blue) and after ITO/APTES modification (green). (**B**) Refractive index n of the ITO layer (blue) and the obtained APTES layer (green) derived from optical modeling of ellipsometric parameters.

ITO and ITO/APTES electrodes were characterized by means of two electrochemical and optical methods, i.e., CV (Figure 3) and ellipsometry (Figure 4).

The results are displayed in Figure 3, where the presence of APTES is shown to significantly reduce the sensor’s conductivity. However, the presence of APTES serves to coat the electrode surface with –NH_2_ groups, which are essential for protein immobilization on the surface.

The experimental curves in Figure 4A show ellipsometric parameters Ψ and Δ dependence on wavelength before and after modification using APTES.

Further, to determine the thickness of the formed APTES layer, an optical model based on regression analysis was applied. This model consisted of a glass substrate and a 168.97 nm ITO layer on top. The ITO layer in the optical model was characterized using ITO optical constants dispersion from the Complete EASE database. The ITO layer thickness was a free-fitting value. After that, the optical model was extended by adding an APTES layer on top of ITO. This layer was characterized using the Cauchy dispersion function with the following fixed parameters: A = 1.546, B = 0.010, and C = 0. The dispersion curves of the ITO and APTES refractive index are presented in Figure 4B. The thickness of the APTES layer was a free-fitting value. After the fitting procedure, it was obtained that the APTES layer thickness was 4.32 nm, which is in good agreement with the literature [50].

Application of electrochemical and optical methods, i.e., CV and ellipsometry, gives interesting insights into the electrode surface properties. The electrochemical method clearly demonstrates that the ITO surface loses primary conductivity due to the APTES. However, the ellipsometric evaluation demonstrates that the layer thickness was only 4.32 nm.

**Figure 5:**
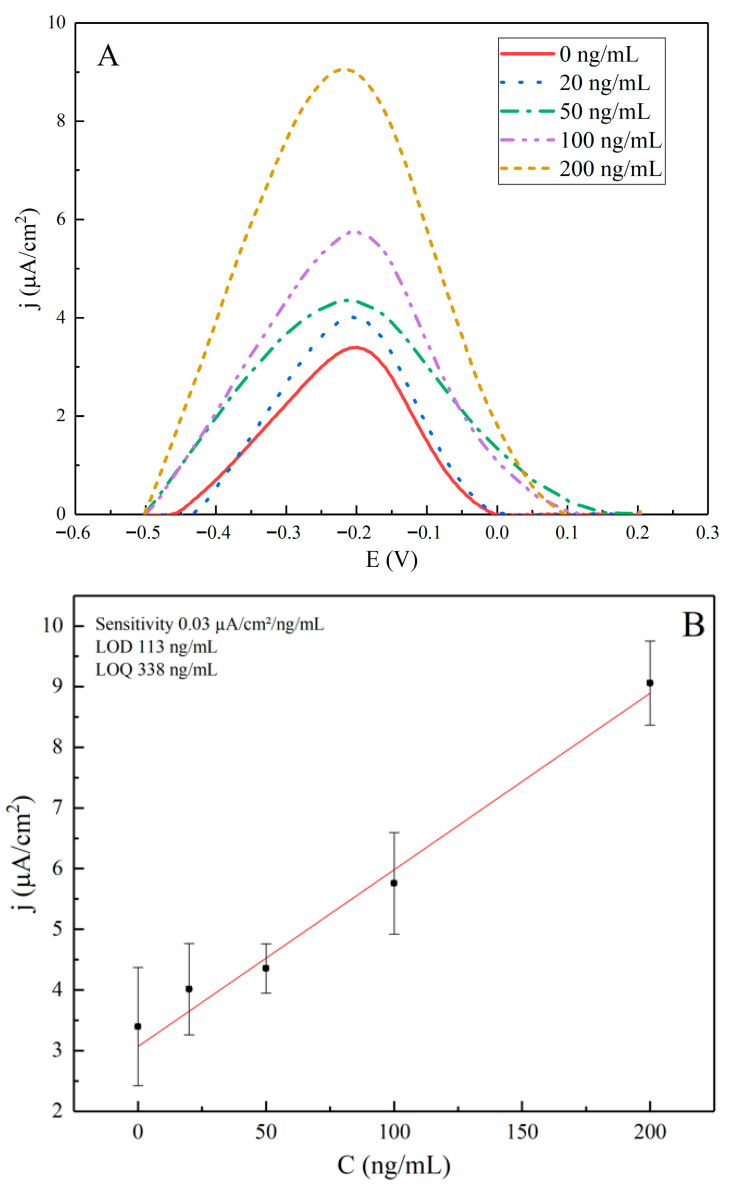
(**A**) Square wave voltammograms with baseline correction of ITO/APTES/SCoV2-rS/anti-rS in the range of concentrations from 0 to 200 ng/mL. Measurements were performed with the following parameters: potential window from −0.6 to +0.3 V, a step size of 0.005 V, a modulation amplitude of 0.025 V vs. Ag/AgCl(KCl_3M_s), and frequency of 20 Hz in a 0.1 M PBS solution. The signal was normalized to the electrode area of 0.196 cm^2^. (**B**) Calibration curves for SWV: current density (j) vs. anti-rS antibody concentration. Error bars represent standard deviations calculated from three independent measurements.

The electrochemical performance of the ITO/APTES/SCoV2-rS/anti-rS biosensor was assessed using SWV across a range of anti-rS antibody concentrations from 0 to 200 ng/mL, as summarized in Table 1 and illustrated in Figure 5A. The measured current density (j) showed a clear increase with rising concentrations of anti-rS, indicating effective binding to the immobilized SCoV2-rS protein.

As shown in Figure 5A, the shapes of the SWV voltammograms differ slightly from each other, with a subtle shift in potential as the concentration of anti-rS antibodies increases. This trend reflects variations in antibody binding efficiency across the tested concentrations and possible inaccuracies during baseline corrections.

The calibration curve (Figure 5B) demonstrates a linear relationship between the j and the anti-rS concentration, with a high quality of linear fit (adjusted R^2^ = 0.98). However, the error bars are quite broad, indicating variability in the measurements.

The calculated sensitivity of the sensor was determined to be 0.03 µA/cm^2^/ng/mL, representing the response per unit increase in antibody concentration. The LOD and LOQ were calculated using the formulas LOD = 3.33 × SD/Slope and LOQ = 10 × SD/slope, where SD is the standard deviation of the blank sample ([anti-rS] = 0 ng/mL). The LOD was found to be 113 ng/mL, and the LOQ was determined to be 338 ng/mL. The relatively high values for LOD and LOQ reflect the variability in the standard deviations observed, which limits the sensor’s ability to detect lower concentrations with high precision.

In our previous studies, we demonstrated that SCoV2-rS was covalently attached through primary amine functional groups on a gold surface, and the formed monolayer was stable and reproducible. Moreover, it has been proven that the impedimetric method and the applied experimental conditions, based on SCoV2-rS covalent coupling, are suitable for the further development of electrochemical biosensors for the serological diagnosis of COVID-19 [51].

The results obtained from the electrochemical characterization of the ITO/APTES/SCoV2-rS/anti-rS biosensor revealed relatively high SDs in j measurements, particularly for blank samples. These elevated SDs contributed to the high LOD observed in this study. A potential reason for this variability may stem from the electrode and biosensor design process, specifically during the surface modification and immobilization steps.

The activation of the ITO surface through argon plasma treatment and subsequent incubation with APTES aimed to enhance the formation of hydroxyl groups, thereby promoting better biorecognition. However, inconsistencies in this surface modification process, such as uneven distribution of APTES or incomplete formation of the SAM, could lead to variations in the binding sites available for immobilizing SCoV2-rS. This lack of uniformity could also explain the observed fluctuations in current density, leading to larger SDs and, consequently, a higher LOD.

Additionally, the biosensor’s design, which incorporates ITO as the working electrode along with the APTES layer for protein immobilization, presents both advantages and challenges. While the choice of ITO provides optical transparency and good electrical conductivity, careful control of surface properties is essential to ensure reproducibility in measurements. The covalent binding of SCoV2-rS to the ITO/APTES surface via GA requires precise conditions to achieve optimal binding efficiency and minimize non-specific interactions.

While various electrochemical methods are available for detecting antibodies against SARS-CoV-2 (Table 2), the application of SWV with ITO electrodes specifically for this purpose is relatively uncommon. This makes our approach quite unique. In comparison to other SWV-based biosensors shown in Table 2, our LOD is higher (the conversion to nM was performed assuming the molecular weight of immunoglobulin G is approximately 150 kDa). For example, screen-printed carbon electrode (SPCE)-based sensors have achieved LODs as low as 47 pM for nucleocapsid protein as the sensing element. However, this method relies on the use of redox mediators. In contrast, our biosensor can detect SARS-CoV-2 antibodies directly from real serum samples without the need for redox mediators, thereby simplifying the measurement process. Furthermore, when comparing our findings to other ITO-based methods, such as those utilizing electrical resistive sensing or photoelectrochemical techniques, our use of SWV for the direct detection of SARS-CoV-2 antibodies stands out as a not widely used approach with comparable LOD.

Despite these strengths, the observed LOD of 113 ng/mL (0.75 nM) highlights a clear need for improvements. Future work should focus on optimizing the surface modification process, refining the electrode design, and exploring alternative strategies for antibody immobilization. By addressing these factors, we aim to enhance the sensitivity of our biosensor and lower the LOD, ultimately improving its applicability for rapid diagnostics.

## 4. Conclusions

In conclusion, the ITO/APTES/SCoV2-rS/anti-rS biosensor demonstrates potential for the direct detection of antibodies against SARS-CoV-2 in real serum samples, providing valuable insights into the evaluation of immune status after COVID-19 infection. The findings highlight the applicability of SWV, marking a unique contribution to the field of electrochemical immunosensors for monitoring concentration of antibodies specific to SARS-CoV-2 spike protein. Continued optimization of the biosensor design and surface modification processes will be essential to enhance sensitivity and improve LOD and LOQ, thereby improving its applicability in clinical settings.

## Figures and Tables

**Table 1:** Analytical data obtained from SWV detection of anti-rS.

[anti-rS], ng/mL	j, µA/cm^2^
0	4.09 ± 0.98
20	3.48 ± 0.75
50	4.07 ± 0.41
100	5.17 ± 0.84
200	8.57 ± 0.69

**Table 2:** Electrochemical biosensors have been previously reported for detecting antibodies against the SARS-CoV-2 virus.

Electrode	Sensing Element	Method	Redox Probe	LOD	
SPCE	Spike glycoprotein	CV, DPV	[Fe(CN)_6_]^4−/3−^	0.27 nM, 0.14 nM	[21]
SPCE	Spike glycoprotein	DPV	[Fe(CN)_6_]^4−/3−^	0.30 aM	[52]
SPCE	Spike glycoprotein	Electrochemical impedance spectroscopy (EIS)	-	0.42 nM	[53]
SPCE	Nucleocapsid protein	EIS, SWV	[Fe(CN)_6_]^4−/3−^	16 pM, 47 pM	[54]
SPCE	Nucleocapsid protein	Chronoamperometry	-	13 pM	[55]
Paper-based SPCE	Receptor-binding domain	SWV	[Fe(CN)_6_]^4−/3−^	6.40 pM	[56]
ITO	Spike glycoprotein	Electrical Resistive Sensing	-	~0.50 nM	[57]
ITO	Spike glycoprotein, Nucleocapsid protein	Photoelectrochemical	-	1.18 nM, 0.65 nM	[58]
ITO	Spike glycoprotein	SWV	-	0.75 nM	This study

## Data Availability

The data presented in this study are available on request from the corresponding author.
